# A Written Constitution: A Case Not Made

**DOI:** 10.1093/ojls/gqab016

**Published:** 2021-05-03

**Authors:** Jo Eric Khushal Murkens

**Keywords:** constitution, written, unwritten, codification, popular sovereignty, territorial constitution

## Abstract

Whether the UK needs a written constitution is a staple of British constitutional debates. Over the years, the fault lines have shifted from whether to incorporate a Bill of Rights to much deeper disagreement with respect to the people and the central power of the state. In this article I neither endorse the conservative case against a written constitution nor argue for the existing constitution to be codified. Instead, I first assess the content of various proposals for a written constitution. I then problematise the process of constitution making by asking not whether the UK constitution should be codified, but by relating the constitution to the people as the authors and to the state as its object.

## 1. Introduction

Should the UK adopt a codified constitution? The question is one that is energetically debated. The argument for a codified constitution seems obvious. It is commonly assumed that written constitutions would tidy-up the existing arrangements. Internal housekeeping would not only bring the UK into line with almost all other states that have codified constitutions (the exceptions are Israel, New Zealand, Sweden and Saudi Arabia), but would also improve the quality of public discussions through better knowledge about governmental processes and institutional arrangements. The central benefit of codification would, therefore, be ‘clarity and definiteness’.^1^

The symbolic 800th anniversary of Magna Carta in 2015 and the ongoing implications of the EU referendum in 2016 have added to the chorus of commentators spelling out the virtues of written constitutions. EU withdrawal had an immediate impact on every aspect of government, from the status of referendums, the relationship between popular and parliamentary sovereignty, the politicisation of the civil service, the contemporary use of prerogative powers, relations between the executive, the legislature and the judiciary, and, finally, to the Union itself. The process of withdrawal has left the unwritten constitution in ‘crisis’,^2^ in a ‘molten’ condition,^3^ and has highlighted the need for a written constitution.

In this article I will assess the arguments that have been advanced in favour of codification, as well as the content of the draft constitutional proposals that have been published over the last 30 years. Most of the proposals satisfy the formal qualities of clarity, concision and coherence. They do so primarily by preserving the existing structures of government in a singular and hierarchical constitutional document for a unitary state. The constitutional proposals are drafted in the language of the rational constitution that articulates the monolithic idioms of sovereignty, ultimate authority, entrenchment, enforceability, uniformity, unity and finality. So far, so modern. But the territorial politics of the UK suggests that codification will not succeed as a ‘completely theorised agreement’^4^ that conveys the clarity of constitutional law, the unity of constituent power and the common identity of a national people. Instead of providing the solution to the crisis, codification begs the constitutional question: it assumes the structures of government, the existence of a people and the integrity of the state when all these issues need to be problematised, interrogated and reconstructed before they can be written down and applied across the UK.

Apart from being an essential piece of good housekeeping, written constitutions are also viewed as a civic covenant to enhance the rationality, humanity and inclusivity of a representative democracy based on popular sovereignty.^5^ In the second section of the article I relate the idea of a written constitution to the people who are meant to authorise it, and to the state which it is supposed to serve. The connections between constitution, people and state are, of course, axiomatic. However, the act of writing a constitution oscillates between contradictory but mutually dependent processes that locate the authority of the people not simply in popular consent, but also in a system of human rights. And whereas constitution making necessarily reinforces the centralised governance structures, in a union state it must also accommodate the decentralised structures with respect to territory, population and government. A civic covenant is an ongoing conversation that strikes a balance between advancing clarity and openness and finding closure. It initiates a never-ending dialectic of mutual construction and ambivalent relations: the constitution constructs the government, the people and the state, which in turn explain the constitution. Those making the case for a written constitution need to provide more information about which aspects are fixed, flexible and foreclosed.

Codification will only succeed as an ‘incompletely theorised agreement’ that recognises ambiguity and lack of clarity, reconciles formally contradictory principles, harnesses opposing dynamics and acknowledges difference. A written constitution should not be a ‘freeze frame’, generated by a still image of the constitution taken at a particular moment. It should instead result from a long exposure to transitional processes of redefinition and renewal. The question should not ask whether it is desirable for the UK to adopt a written constitution, but how the UK should be reconstituted.

## 2. Constitution and Codification

Constitution making is a political act. Choosing the basic principles of constitutional design, endowing the document with legitimacy and ensuring its utility as a political artefact combines ‘science, art and craft’.^6^ But constitution making, especially in a country like the UK, would necessitate deep changes for the legal and political culture,^7^ especially if the constitution replaced Acts of Parliament as the highest source of law. The doctrine of parliamentary supremacy, together with the rule of law and conventions, forms the backbone of the UK’s unwritten constitution. It has always been and remains a totemic issue in constitutional debates. As the highest source of law, Acts of Parliament may violate international law and fundamental rights and repeal constitutional statutes at will. The UK Supreme Court may have attempted to disaggregate parliamentary supremacy by intimating that, in the context of rights, the doctrine is ‘no longer … absolute’,^8^ but in the context of the devolved governance structures, the same court has deemed Westminster’s power to legislate for Scotland and Wales to be ‘undiminished’.^9^ Martin Loughlin and Stephen Tierney argue that this dominant institutional conception of absolute legislative authority is a ‘primitive view’ that ‘rests on an inchoate appeal to the need for Westminster to hold on to untrammelled power [which] is inadequate and must be jettisoned’.^10^ This primitive view is most incompatible with, and therefore most threatened by, the adoption of a written constitution. If it is retained, the UK will remain ‘incapable of being constitutionalised’.^11^ If it is repudiated, the culture of the matchless, flexible, political constitution will have been corrupted. Rooted in conservatism^12^ and anti-rationalism,^13^ and sceptical of the constitutional responsibilities of courts,^14^ opponents view the idea of a written constitution as ‘unnecessary, undesirable and un-British’.^15^ As Roger Scruton has observed:

Conservatives in the British tradition are heirs to an island culture, in which custom prevails over reason as the final court of appeal … When interrogated as to the justice or reasonableness of any particular part of their inheritance—be it the common law, the monarchy, the nature and workings of parliament, the Anglican Church and its nonconformist offshoots—they tend either to shrug their shoulders, asserting that this is how things are because that is how they were, or else they take refuge in irony and self-mockery, confessing to the absurdity of a system whose principal merit is that nobody knows why it exists, and hence nobody quite knows why it shouldn’t.^16^

The ‘era of constitutional reform’ since 1997 has been formed by landmark legislation and institutional changes.^17^ Although these changes indicate a lack of faith in the traditional constitution, they also demonstrate an absence of political will for a new constitution. The process was not guided by clear mechanisms or overarching principles. Some decisions, eg on continued membership of, and withdrawal from, the European Union (1975; 2016) and on devolution and mayoral referendums (since 1998), involved plebiscites. Some legislation, like the Human Rights Act 1998, was preceded by a government White Paper explaining the policy.^18^ Others changes, such as the reform of the office of the Lord Chancellor (2003) and the inauguration of the UK Supreme Court (2009), took place even without public consultation. Arguably, the domestic reforms are linked by the gradual embracing of the doctrine of separation of powers.^19^ However, separation of powers is ‘a newfound religion, not much revealed in Government scripture before 2003’.^20^

Over the past 50 years,^21^ a number of judges, scholars, politicians and think tanks have put forward their arguments for codification and, in a handful of cases, have taken the time and effort to produce a draft constitution. On the conservative side, Lord Hailsham called for a new constitution and Bill of Rights in the 1970s.^22^ On the liberal side, Lord Scarman gave lectures over three decades in support of a written constitution.^23^ Charter 88, a pressure group, backed constitutional and electoral reform with the support of Gordon Brown,^24^ who supported a written constitution also as Prime Minister.^25^ In 1991, the Institute for Public Policy Research (IPPR) produced a 136-page long ‘constitution’, which consisted of 129 articles and six schedules.^26^ In 1993, Tony Benn wrote a radical proposal for a reconstitution of the UK as a Federal Commonwealth with an elected Head of State.^27^ Oxford students drafted a constitution, under supervision, in 2006,^28^ and Richard Gordon published a draft constitution with 248 clauses in 2010.^29^ In the same year, Vernon Bogdanor and Stephen Hockman led a ‘constitution working group’ that identified problems, questions, and options with respect to codification.^30^ The debate intensified in the run-up to the 800th anniversary of Magna Carta in 2015.^31^ The House of Commons Political and Constitutional Reform Committee (HCPCRC) set out three models of a codified constitution (a declaratory Code, a Consolidation Act and a Written Constitution) and, following a nationwide consultation, found broad popular support for codification.^32^ From 2013 to 2015, the LSE’s Institute of Public Affairs crowd-sourced a 30-page long ‘People’s Constitution’.^33^ Since the referendum on EU withdrawal in 2016, a steadfast number of lectures,^34^ debates,^35^ broadcasts,^36^ newspaper articles,^37^ reports^38^ and publications^39^ have corroborated the growing concerns with codification. In the December 2019 General Election, parties as diverse as the Liberal Democrats, the Brexit Party, the Green Party and the Alliance Party of Northern Ireland advocated a written constitution for the UK.^40^

The constitutional proposals give rise to two sets of questions. First, what is the purpose of codification? Is it to clarify the rules of government? Or is it to regulate the more intricate relationship between the people and the state? Secondly, how ‘un-British’ are the draft constitutional proposals? Would a written constitution reflect ‘the soul of a nation’ by faithfully translating the UK’s cultural, political and legal tradition into written form? Or would codification fire up the ‘engine of social transformation’ and reconstitute the nation by creating a framework for a different political order and an aspirational future?^41^

The primary function of a constitution is to serve as an instrument of government. Enabling rules set out a formal framework for the basic rules that regulate the activity of governing within a state and authorise decision making. Nominal or organisational constitutions^42^ are limited to

the set of laws, rules and practices that create the basic institutions of the state, and its component and related parts, and stipulate the powers of those institutions and the relationship between the different institutions and between those institutions and the individual.^43^

The primary concern of all the draft proposals lies with clarifying the structures of government and fundamental rights. Most of them would preserve the hereditary monarchy, the Church of England, the adversarial political system and, worryingly, the doctrine of parliamentary supremacy.^44^ Most of them display no enthusiasm for higher-law constitutional limits on Parliament’s law-making ability. Although the most recent Written Constitution by the HCPCRC confers a power on the UK Supreme Court to review the compliance of Acts of Parliament with the Constitution, it only empowers the Court in cases of non-compliance to ‘make a declaration of unconstitutionality that does not invalidate the statutory provision in question’.^45^

The conservative nature of the codification debate in the UK is intensified by the conservative nature of the codification process itself. Comparative experience suggests that creating a constitution is constrained by collective choices (the goals, the mechanisms),^46^ but also by the imagination of the drafters, which ‘is framed by what already exists’.^47^ The modesty of the British debate creates a tension between constitutional replacement and constitutional conservation. The authors of the constitutional proposals typically set out to ‘change the basis of the Constitution’,^48^ ‘to chart a new course’^49^ and to replace the ‘out-dated dogma’.^50^ But most of the constitutional prototypes end up displaying a cognitive preference for the central features of the unwritten constitution, albeit in codified form. The comparatively progressive proposal by the IPPR in 1991 has to concede that ‘much of the content is in the best (or worst) tradition of gradualism. The main features of the present Constitution are left more or less intact.’^51^ Although Richard Gordon’s draft breaks ground by empowering the people and subjecting Parliament to the constitution, in all other respects he aims ‘to collate in written form much of our existing informal constitution’.^52^ If the purpose of codification is to conserve constitutional choices that already exist, would the adoption of a written constitution still be ‘a pre-eminently political act’?^53^ Would it even be a meaningful one? In the following paragraphs I will assess the reasons given in support of codification.

If one were to believe the literature, the main benefit of having a written constitution would lie in improving the quality of the debate by generating knowledge and clarifying the laws of the constitution. Education, according to Robert Blackburn, ‘is one of the strongest arguments for having one’.^54^ A constitution that sets out the basic rules, procedures and institutions of government would act as a guide for all public office holders, as well as ‘a reference point in everyone’s upbringing and education’.^55^ Opinion polls support the need for political education and legal clarity. Ipsos Mori found in 2008 that only 20% of the respondents believed they knew the UK’s governing arrangements ‘very well or fairly well’.^56^ In 2018, almost two-thirds (65%) of respondents in a YouGov poll wanted ‘a written constitution providing clear legal rules within which government ministers and civil servants are forced to operate’.^57^ In 2019, that percentage jumped up to 72% on a very similar question in a ComRes/Daily Express poll.^58^ In 2015, the HCPCRC acknowledged the clear popular support for a written constitution in opinion polls as well as its ‘beneficial educative effect in society, for young persons at school and the country in general’.^59^

However, public edification is not the *purpose* of constitutions.^60^ A constitution may have an educational *effect*, but that is very different from it being an objective. In addition, it is far from clear that a comprehensive constitutional code would result in a ‘pocket constitution’ (a printed copy that fits in a pocket or purse)—a metonym for ease of reference and accessibility. In 2013, the Comparative Constitutions Project pulled together the UK’s extant constitutional statutes from Magna Carta 1297 to the Fixed-term Parliaments Act 2011.^61^ Without including judicial decisions, prerogative powers, conventions or parliamentary custom and governmental practices, the final document exceeded 700 pages and consisted of 225,000 words—almost the length of James Joyce’s *Ulysses*. India usually tops the league table for the longest codified constitution in the world, at 146,000 words spread over 444 articles in 22 parts with 118 schedules. But, as James Melton and his colleagues, who oversaw the Project, note, the UK has the ‘longest and, arguably, the most complex constitution in the world’.^62^

The HCPCRC’s proposals fare better in that respect. Their Constitutional Consolidation Act consists of 231 articles over 239 pages, whilst their Written Constitution contains 53 articles over 74 pages. In terms of length and style, especially the Written Constitution purports to be more of a ‘layman’s’ (as opposed to a lawyer’s) document, as Franklin Delano Roosevelt called it in dated terms, in that it strives to ‘enable everyone to know what the rules and institutions were that governed and directed ministers, civil servants and parliamentarians in performing their public duties’.^63^ It does so by clarifying the powers of the Crown, the government’s prerogative powers, the legal status of referendums and human rights, the role of the devolved administrations and the competences of the devolved parliaments. But mainly it succeeds in doing so by maintaining the status quo: ‘we deliberately did not set out to propose constitutional codification with radical constitutional reform’.^64^

The written form and the organisational substance do not complete the concept of constitution. According to Giovanni Sartori, the form is only a means, when ‘what really matters is the end, the *telos*’,^65^ namely the purpose of the framework, which is the prevention of over-concentration and arbitrary power. For the past 250 years, this *telos* has placed limitations on government: ‘it is undeniable that the whole of the American tradition has understood “constitution” as a means for “limited government”’.^66^ In the standard account, governmental decision making is ‘disabled’ by subjecting the political decision-making process to substantive requirements (Bill of Rights) and by the existence of procedural obstacles to constitutional amendment (higher voting thresholds, referendum requirements). However, it is important to note that enabling rules and disabling rules are complementary, not contradictory. Drawing on John Searle, Stephen Holmes argues that a modern constitution does not limit government, but it does enhance democracy. Searle and Holmes distinguish between regulative rules which control a pre-existing activity (eg ‘no smoking’) and constitutive rules which initiate and enable an activity (eg ‘bishops move diagonally’).^67^ The political and legal systems consist of both enabling and disabling rules. The linchpin is the constitution, which legitimates the exercise of state power by providing ‘political solutions for the problem of the self-reference of the legal system and legal solutions for the problem of the self-reference of the political system’.^68^

Advocates for a written constitution and a Bill of Rights do not view enabling and disabling rules as complementary. But nor do they fully embrace the *telos* of limited government. The debate about a Bill of Rights has in the past acted as a proxy for a written constitution, and it sheds more light on the projected relationship between the people and the state. The government’s Green Paper in July 2007 envisaged a ‘British Bill of Rights and Duties’.^69^ A government commission referred to the existing framework of ‘rights and responsibilities’.^70^ As is clear from the qualifying concepts of duties and responsibilities, from the government’s perspective rights ‘must be exercised in a way that respects the human rights of others’.^71^ In 2008, the Joint Committee on Human Rights unequivocally recommended the adoption of a ‘Bill of Rights and Freedoms’ without, however, recommending entrenchment against future amendment or repeal by requiring a special parliamentary majority or referendum requirement. ‘In our view such forms of entrenchment are not compatible with our tradition of parliamentary democracy which has carefully preserved the freedom of each Parliament to legislate according to its view of the public interest.’^72^ Once more, parliamentary sovereignty is viewed as a value greater than rights protection, stifling the significance of codification from the outset. The Joint Committee also formed the view that, although economic and social rights, such as health, education and housing, should be included in a Bill of Rights, individual litigants would not be able to enforce those rights against the government or any public authority.^73^ Whereas in other contexts human rights assert inspirational values and aspirational principles as universal, inalienable, indivisible and refer to all persons as human beings, not as citizens,^74^ ‘British’ human rights would be qualified by duties, responsibilities and the enhancement of ‘our liberties’ for ‘our citizens’ in ‘our country’.^75^

Human rights are supposed to be justifiable uniquely and ‘exclusively from a moral point of view’,^76^ as opposed to ethical or pragmatic considerations. By way of contrast, the British proposals justify human rights on grounds of their instrumental value ‘to ensure that the system works better to protect the individual against the powerful’.^77^ It is true that human rights offer functional protections from arbitrary government on an individual basis through disabling rules. But that tells only one half of the story, making it still a ‘very British’ story. Rights are also enabling rules that facilitate the communicative conditions for ‘democratic opinion- and will-formation that justify the presumption that outcomes are rationally acceptable’.^78^ This is the point about their objective moral justification. The concept of human rights is oriented towards the common good, which is constructed through the discursive articulation of individual rights into a comprehensive system of public rights. The legally guaranteed protection of the *private* autonomy of the individual may be the focal point of lawyers, politicians and society in general. But conceptually, it is a condition for the *political* autonomy of citizens which is realised using reason. In the same way that enabling and disabling rules are complementary, the two sides of human rights are also interdependent.^79^

Almost without exception, current proposals for codification would not remodel the structures of government but affirm them. Codification would not involve a caesura with the existing order, but produce ‘greater clarity, wider and deeper dispersal of power, and a firmer more enforceable set of principles and rules’.^80^ Codification would protect rights, but not alter the current scope of rights protection. A written constitution would systematise historical social practices, not equate to an act of rational design. In short, as things stand, constitution making would be neither a political nor a cultural act. The British debate has successfully depoliticised the arguments for codification and neutralised the possibility of cultural reform. The options for the drafters are bounded by a status quo bias and by constitutional imaginations that are steeped in British political culture.

Men make their own history, but not of their own free will; not under circumstances they themselves have chosen but under the given and inherited circumstances with which they are directly confronted. The tradition of the dead generations weighs like a nightmare on the minds of the living.^81^

The draft proposals purport to clarify the rules of government to bring about a new constitutional paradigm. The proposals arguably deliver on the former, but they do not deliver on the latter. Clarity and certainty are compatible with conservation and continuity, but do not on their own amount to replacement and reform. Moreover, most of the proposals would serve as instruments of government and as nominal constitutions. Fundamental questions, such as how to construct the unity of a people or how to serve the UK as a union state, are conspicuous by their absence. It is to these questions that I turn to next.

## 3. Constitution and the People

There is a near-universal understanding that, to be legitimate, the authority for a constitution must derive from the people of the state concerned—an understanding that stems from the anti-colonial movements in revolutionary North America and has subsequently migrated around the world.^82^ However, not all proposals for a codified UK constitution are drafted in the name of ‘We the People’.^83^ This is unsurprising: the role of an instrument of government is to clarify the rules and institutions under which ministers, civil servants and parliamentarians operate, not to wax lyrical about British values and the character of the British people. This stands in contrast to Thomas Paine’s dictum that ‘the constitution of a country is not the act of its government, but of the people constituting its government’.^84^ The draft preambles by the IPPR in 1991, the LSE Institute of Public Affairs and Richard Gordon were drafted in the name of the people.^85^ If the written constitution became the collective civic covenant of the British people, and not a simple reflection of the rules of government, what version of ‘popular sovereignty’ would authorise it? In this section, I discuss three commonly used ideal types of popular sovereignty (*patris*, *ethnos*, *demos*).^86^ All of them are well-known, none of them were conceived in a context of universal suffrage and none of them are workable for the plurinational UK. This is also problematic for politicians who invoke the ‘will of the people’ as a mandate for policy choices.^87^ There is a solution, but before I propose it, we need to clear away the assumptions about popular sovereignty.

The strongest conception of popular sovereignty amalgamates the democratic legitimacy of state power with the constituent power. We need to distinguish between the ‘populus’, or the population as such, and the political existence of a people. The strong conception presupposes an antecedent people that exists as a political unit prior to the constitution. That ancestral nation is *patris* (homeland). The state is not the result of the social contract, itself ‘an exercise of the imagination’,^88^ but an actual entity with history, institutions, culture, language, ethics, an economy and art. It is held together, according to Georg Wilhelm Friedrich Hegel, not by force or fear, but by ‘the basic sense of order which everyone possesses’.^89^ In other words, the general will manifests itself as concrete, social consensus. The people can write any constitution they want and retain a power to withdraw support and give themselves a new constitution at any time.

The strongest conception can be detected in political discourse, whether it is a ‘British Bill of Rights’, ‘British values’, ‘bringing rights back home’ or PM Theresa May’s interpretation of the 2016 referendum result as a decision by ‘the people of the United Kingdom’ to ‘restore, as we see it, our national self-determination’.^90^ However, the construction of public ownership in this way does not work, given the size and ethnic composition of the UK population and the presence of different territorial identities. A range of people from different ethnic backgrounds live in England, Scotland, Wales and Northern Ireland. ‘Britain’ is at once a territorial and political unit that excludes Northern Ireland and an ‘imagined community’,^91^ ie a socially constructed and psychologically represented social system in which ‘being British’ may be relevant and may be mobilised in some situations but not in others.^92^*Patris* is also undesirable and unnecessary. Its undesirability stems from its absolutist quality. *Any* measure sanctioned by the people would be valid on the ground that it articulates the homogeneous values and a shared identity. Popular sovereignty would be a like-for-like replacement for parliamentary sovereignty. In addition, a constitutional document is unnecessary for a substantively homogeneous people with shared values and a common identity. If the political unity of such a people is strong, then it does not require the assistance of a constitutional document (‘gentlemen do not need to put their word in writing, it is enough simply to give one’s word’^93^). If the political unity is weak, because the values and identity are diluted, then the fertile ground on which popular sovereignty flourishes is degraded, and the integrity of the people as *patris*, as an *a priori* decision-making unit, disappears.

The second ideal type of sovereignty contains no antecedent people and no prior right to political authority. For Jean-Jacques Rousseau, the creation of the constitution *ex nihilo* is a ‘civil act’. It requires prior deliberation and, crucially, unanimity. Majoritarian support is insufficient. The organising principle here is *ethnos* (nation). A legitimate political order requires an act of self-constitution by the people, which means that the minority must give their free consent if they are obliged to obey.^94^ ‘Every man being born free and his own master, no one, under any pretext whatsoever, can make any man subject without his consent.’^95^ The constitution is, therefore, a pact of association (‘the act by which a people becomes a people’),^96^ not a pact of submission (in which people elect leaders).

Although Rousseau was ‘the champion of popular sovereignty’,^97^ his conception self-evidently does not apply to the UK. Advocates of codification are not proposing that the people authorise a new social order in a revolutionary moment. Moreover, Rousseau’s requirement for unanimity (and the death penalty for dissenters from the unanimous body) for the adoption and abrogation of a constitution is a non-starter.^98^ His advocacy of a strict amendment formula and of the procedural entrenchment of fundamental laws is designed to ‘strengthen the constitution’^99^ and to prevent the popular sovereign from enacting a new constitution in the future. These requirements are difficult to reconcile with the codification debate in the UK.

In the third ideal type, popular sovereignty inheres in a constitutional framework of representative government, political equality and participation rights for the *demos* (people). For Immanuel Kant, consent by the governed is the only source of governmental legitimacy. But what kind of consent? According to Kant, actual consent by the people does not constitute a valid criterion for the substantive legitimacy or correctness of laws.

This is the test of the rightfulness of every public law. For if the law is such that a whole people could not *possibly* agree to it (for example, if it stated that a certain class of *subjects* must be privileged as a hereditary ruling *class*), it is unjust; but if it is at least *possible* that a people could agree to it, it is our duty to consider the law as just, even if the people is at present in such a position or attitude of mind that it would probably refuse its consent if it were consulted.^100^

Instead, it is the hypothetical will of a reasonable people that validates laws and guides lawmakers. But this creates a problem for democratic states: how can a political system call itself democratic in the absence of actual participation and consent from ‘the will of the entire people’, which is the only available strategy to legitimise governmental activity? Kant needs to marry hypothetical consent with an element of actual consent. Prior unanimous consent is, therefore, required for the principle of majoritarian decision making. Substantive unanimity is illusory—people will inevitably disagree on the substantive correctness of a decision. It is, therefore, imperative that all citizens unanimously consent to the principle of majoritarian decision making: ‘Thus the actual principle of being content with majority decisions must be accepted unanimously and embodied in a contract; and this itself must be the ultimate basis on which a constitution is established.’^101^

Kant does not discuss the people as a constitutive power, as in the other two versions of popular sovereignty, but as a constituted power. ‘A state (*civitas*) is a union of a multitude of men under laws of Right.’^102^ Within the lawfully constituted state, the legislative authority belongs ‘only to the united will of the people’, which, as the source of all rights, ‘*cannot* do anyone wrong by its law’.^103^ But the will of the people exhausts itself in the competences that are defined in the constitution. There is no antecedent people separated from the constitution, and the people do not enjoy a residual, sovereign power to overthrow the constitution at will. The idea that the people are the historical authors of the constitution is exposed as a myth,^104^ and the legitimacy of collective decision making cannot be reduced either to the voluntariness of consent or to the reasonableness of an agreement.

For all its advances on the other two versions of sovereignty, it is not clear that the Kantian *demos* is a perfect match for the UK. The popular sovereign, the *demos*, is umbilically tied to the constitution, which means that the people cannot abrogate and replace their constitution while retaining their political existence and identity.^105^ That does not sound like a winning formula for a people used to constitutional flexibility and unbridled freedom of action. Moreover, on every model of sovereignty, the discussion of ‘the people’ is complicated by conceptions of nationhood that are fixed. *Patris*, *ethnos* and *demos* each generate a stable image of ‘the people’, but with different interpretations in relation to general will, unanimity and majoritarianism.

But there is a more important reason for the complication. The revolutionary claim that all state power derives from the singular source of the people has a destabilising quality. The democratic principle ostensibly authorises the lawmaker not just to set up a constitution, but also to violate its own basic laws. Pure voluntarism puts paid to the notion of the constitutional state if whatever measure is enacted in the name of the people is law. The task of stabilising state power falls to disabling rules which, by placing strategic limits on governmental decision making, prevent a democracy from abolishing itself.^106^ Stability, in turn, begets legitimacy, which constitutional regimes have acquired over time by withdrawing core competences from the voluntarism of a sovereign people and by anchoring the process of constitution making in the rationality of universal human rights.^107^ In other words, the legitimacy of government stems from the dual commitment to the democratic self-determination of citizens and, as discussed above, to the articulation of individual rights into a comprehensive system of rights. Jürgen Habermas’s discourse theory harnesses the dual commitment to popular sovereignty and human rights, free consent and reasonable agreement, will and reason. It allows him to view the people as neither constitutive (*patris*, *ethnos*) nor constituted (*demos*), but as co-constitutive with a universal ideal of rights protection.^108^ ‘What unites a nation of citizens as opposed to a *Volksnation* [*patris* and *ethnos*] is not some primordial substrate but rather an intersubjectively shared context of possible understanding.’^109^

The dialectical relationship between popular sovereignty (voluntarism) and human rights (rationalism) as sources of legitimacy is not discussed in any of the three conceptions that provide the deep context for constitution making and which have been discussed in this section. Nor is it expressed in any of the draft constitutional proposals for the UK. References to ‘We the People’ in the drafts are either formulaic or absent. Although all the draft constitutions now protect civil and political rights as a matter of course, which marks a change from previous generations when the case for rights needed to be made,^110^ their inclusion is still cautious. The scope and degree of human rights protection never exceeds those provided by the European Convention on Human Rights. As discussed earlier, the Bill of Rights envisaged by the Joint Committee on Human Rights in 2008 would not have been entrenched and would not have allowed individuals to enforce economic and social rights against public authorities. This is acceptable for a constitution as an ‘instrument of government’. A ‘people’s constitution’, however, involves a symbiotic relationship between popular sovereignty and human rights, and not tentative references to an amorphous people and the lowest possible inclusion of civil and political rights as a compulsory accessory.

## 4. *Constitution and* pouvoir constituant mixte

In addition to the dialectic between popular sovereignty and human rights, the present-day context of the UK creates its own dynamic relationship between the centre and the regions. That dynamic affects the criteria of statehood itself (government, population, territory), which, in turn, makes the UK an uneasy reference point for a single codified document. ‘Government’ is destabilised by the devolved regional legislatures and governments in Edinburgh, Cardiff and Belfast, which have ‘unsettled’^111^ the UK’s constitution. ‘Population’ searches for a demotic entity that is difficult to identify in a state with multiple designations,^112^ four regions and six territorial identities (British, English, Irish, Northern Irish, Scottish and Welsh). ‘Territory’ is brought into question by the UK’s legal commitment to the possibility of Irish unification in section 1 of the Northern Ireland Act 1998,^113^ as well as by the ongoing discussions about a second Scottish Independence referendum,^114^ by a Labour-led Welsh government that refers to the UK as a ‘voluntary association of nations’ based on ‘popular sovereignty in each part of the UK’^115^ and by the presence of a nationalist party talking up the possibility of Welsh independence.^116^ How should the constitution be drafted if the statehood criteria are less well defined than is usual? How can the process of codifying the constitution gain the support of the non-English regions and the loyalty of their populations? How can codification strengthen the Union?

According to conventional domestic constitutional interpretation, Parliament is the obvious body to write and ratify a constitution. It acts as the constituent assembly (it can ‘shift the basis of the constitution’) and as the legislative assembly (it enacts ordinary laws).^117^ It is possible for legislatures more generally to become constituent assemblies for that purpose.^118^ However, three recent interventions suggest that the constitution should not be written by Parliament. In 2012–13, the HCPCRC conducted an inquiry into the need for a UK-wide constitutional convention. The inquiry did not focus on a written constitution, which was the subject of a separate inquiry,^119^ but examined the interaction between the increasingly devolved parts of the UK and considered the future constitutional structure of the UK.^120^ In debating questions relating to the remit, composition and timing of the convention, the inquiry opened up extra-parliamentary avenues to discuss future constitutional change. The Chair of the Committee, Graham Allen MP, even introduced the Constitutional Convention Bill in the House of Commons, but it did not progress past first reading.^121^

Two further interventions came from academics. Bruce Ackerman, writing before the pandemic of 2020, calculated that MPs would be ‘so overwhelmed with the challenges of managing Brexit over the next few years that they will have little time left for anything else’.^122^ He, too, makes the case for a special Constitutional Convention, which should be elected by Parliament under ‘closed list proportional representation’, ‘debate the long-term, big picture issues’ and be given a reasonable deadline to draft a proposal to be put before the British people in a referendum.^123^ Finally, Jeff King’s argument for a written constitution is premised on the position that ‘the people’s representatives should participate in the writing of the fundamental laws of the community’.^124^ He proposes that the constitution be authored by a constituent assembly, ie a special-purpose body to draft a constitution. It should consist of citizens, electors and possibly residents; at any rate, ‘it must represent the people by being “representative, informed, and effective”’.^125^

The problem with a UK-wide Convention is that it adopts a unitary conception of the British people. Although King is careful to dissociate his use of the people from *patris* and *ethnos* (‘a sovereign collective identity whose pronouncements for the people can persist over time’) and from *demos* (‘a distinct and unitary Will of the People determined by majoritarian voting procedures’),^126^ he still views ‘those living in Britain’^127^ as a decision-making unit. While Ackerman acknowledges ‘dilemmas generated by Irish, Scottish, and Welsh demands for home rule’, his solution for ‘the British people’ (which consists in the creation of a new national holiday, Deliberation Day, to encourage voters to discuss the draft constitution a couple of weeks before it is put to them for approval in a special referendum) is distinctly unitary.^128^

A UK-wide Convention assumes a pre-constitutional consensus on the identity of ‘the British people’ as well as shared normative commitments in relation to the state even if those commitments are implemented differently and asymmetrically in practice. No thought is given to a special role for Scotland, Wales and Northern Ireland in the constitution-making process or to their future status in a reconstituted UK. Arguing for a written constitution on the assumption of an ongoing commitment to the Union from the non-English regions represents a leap, and arguably even a lapse, of faith. Political discourse is already marked by policy divergence, by constitutional disagreement (Sewel Convention) and by strong regional parties, especially the Scottish National Party. Even a representative convention or assembly may not suffice to produce the societal consensus required for a stable outcome.

More importantly, the English doctrine of parliamentary sovereignty, which is still asserted as a Unionist principle,^129^ now competes against a Celtic doctrine of popular sovereignty. The principle of consent migrated from the Northern Ireland context^130^ to the opening provisions of the Scotland Act 2016 and the Wales Act 2017, which protect the respective regional parliament and government from being abolished ‘except on the basis of a decision of the people of Scotland [or Wales] voting in a referendum’.^131^ This development raises questions for the legitimacy of constitution making. Would the written constitution derive its authority from a single community of citizens? Or would it represent the peoples of the non-English regions as sources of authority and subjects of legitimacy?

To repeat a point made earlier: to be legitimate, the constitution must derive from the original authority of a sovereign people. As a unitary concept, however, the category of the British people is cumbersome, dominated as it is by the size of the English population, which can always outvote the residents of Scotland, Wales and Northern Ireland. By way of contrast, European democracy theory has developed a new account of political community for the European Union called *pouvoir constituant mixte*. It is not intended as an abstract constituent power for an original founding moment, but as a rational reconstruction of the democratic character of the European Union, which consists of the citizens of the European Union and of the Member States.^132^ For those reasons, *pouvoir constituant mixte* resonates in the UK as well. Every citizen participates in the democratic opinion and will-formation processes as a dual subject: as an *individual* British national and as a *member* of one of the four regions.^133^ Additionally, the *pouvoir constituant mixte* honours a dialectic that is missing from *patris*, *ethnos* and *demos*. It saves the constituent power of the British people through disaggregation and then through reconstruction, allowing it to become not the presupposition, but the aspiration and normative reference point of the constitution. Whereas the unitary British people cannot reasonably be presupposed as the authors of a unitary constitution, the hybrid conception of *pouvoir constituant mixte* could legitimately serve as the basis for a constitutional debate.

Empirically, the relevance of the *pouvoir constituant mixte* for the UK is borne out. Many UK nationals are already comfortable with being members of two *demoi* at the same time. On the Moreno scale,^134^ a majority of English participants identify as English (80%) and British (82%).^135^ A majority of Scots identify as Scottish (84%) and as British (59%).^136^ Just under half of people born in Wales identify either entirely as Welsh (21%) or more Welsh than British (27%), and a similar size identify either as equally Welsh and British (44%) or more British than Welsh (5%).^137^*Pouvoir constituant mixte* is of particular relevance in Northern Ireland, where the Belfast/Good Friday Agreement 1998 recognises ‘the birthright of all the people of Northern Ireland to identify themselves and be accepted as Irish or British, or both, as they may so choose’.^138^ However, according to two recent and separate polls, fewer than half the people identify as predominantly British,^139^ with the majority identifying as Irish, Northern Irish or European.^140^

The possibility of disintegration and dissolution would shape the process of constitution making like the sword of Damocles. The cost of loyalty (integration, assimilation) is too high for the non-English regions, and the cost of exit (Irish unification, Scottish independence) is too high for the Unionist majority. Both options would increase polarisation, regionally as well as nationally. If the UK is to codify its constitution, it will need to accommodate the voice of the non-English regions with favourable terms and conditions. Unfavourable terms and conditions will force them to consider alternative options.

## 5. Conclusion

The literature on codification assumes the integrity of the UK, presupposes the constituent power of the British people and takes the loyalty of the non-English regions for granted. The draft constitutional proposals codify the existing devolution settlement, but they do not seize the moment to reimagine the UK. Since Northern Ireland and Scotland have potential exit routes, their voice in the process of constitution making and operations of state building requires special attention. With the Wales Act 2017, Wales drew level with Scotland in terms of devolved governance arrangements, so it should enjoy parity of status even without a credible exit strategy. It would be a missed opportunity if the codification process preserved a random constitutional moment in time, rather than considered ways of strengthening the Union, like federalism, regional autonomy and *pouvoir constituant mixte*. In the long run, the famously flexible UK constitution will need to morph into an ‘incompletely theorised agreement’ with multilateral, rapid and dynamic response mechanisms to deal with unpredictable changes, not hold fast to centrality, clarity, certainty, rigidity and finality.

While I have expressed here my concerns about the desirability of codifying the existing UK constitution, I have at the same time offered no defence of the institutions, conventions and practices of the unwritten constitution. I am, therefore, not convinced by the attempts to convert the unwritten constitution into a written one. Nor am I against codification as a matter of principle. I am simply not persuaded by the suitability of the globally dominant paradigm of liberal ‘AngloEurocentric constitutionalism’.^141^ The clarity and certainty of the monolithic constitution do not suit the eclectic power structures or the multiregional, plurinational and evolving nature of the UK.

One thing is certain: a constitution that consciously tries to square the circle between the centralised state apparatus and the cleavages wrought by the territorial constitution and the politics of policy divergence would have to be written. To do so, the drafters of an accommodationist constitution would need to study the literature on conflict resolution, power-sharing and consociational techniques rather than the canon of rational, liberal, nation-state constitutions. The accommodationist state would contain multiple public identities and provide for a form of constitutional pluralism that ‘acknowledge[s] the legitimacy of every other [normative order] within its own sphere, while none asserts or acknowledges constitutional superiority over another’.^142^ Accommodation requires provisional solutions, ambiguity, incremental strategies, deferral mechanisms and alternative institutional arrangements that can manage inconsistency as part of a strategy of coexistence.^143^ Such an approach builds capacity for a constitutional dialectic that consists of mutual construction, ambivalent relations and opposing dynamics which, as I have shown, exist in relation to government, the people and the territorial constitution.

Instead of educating citizens, the process would be a steep learning curve for the legal experts and political representatives charged with drafting the constitution. Instead of clarifying the existing UK constitution (all the attempts show that it can be done for an essentially unitary state), a collective effort is required to reimagine and redefine the UK as a state for which there is no precedent or template. For these reasons, the UK should not adopt one of the proposed written constitutions. It should be reconstituted.

## Footnotes

1 AC Grayling, ‘Why the UK Needs a Written Constitution’ in DJ Galligan (ed), *Constitution in Crisis: The New Putney Debates* (IB Tauris 2017).

2 Galligan (n 1).

3 Andrew Blick and Peter Hennessy, ‘Brexit and the Melting of the British Constitution’ (The Constitution Society 2019).

4 Cass R Sunstein, ‘Incompletely Theorized Agreements’ (1995) 108 Harv L Rev 1733.

5 Richard Gordon, *Repairing British Politics: A Blueprint for Constitutional Change* (Hart Publishing 2010); Elliot Bulmer, ‘Do We Need a Written Constitution’, debate between Elliot Bulmer and Paul Bickley (Theos Think Tank, 24 October 2019): ‘We need a restored civic covenant to bind ourselves together as a political community under common rules for the common good.’

6 Daniel Elazar, ‘Constitution-Making: The Pre-eminently Political Act’ in KG Banting and R Simeon (eds), *Redesigning the State: The Politics of Constitutional Change in Industrial Nations* (Macmillan 1985) 233.

7 Sotirios A Barber, *Constitutional Failure* (University Press of Kansas 2014) xvii: ‘constitutional reform must therefore be nothing short of cultural reform’.

8
*R (Jackson) v Attorney General* [2005] UKHL 56, [2005] 1 AC 262 [104]; also [107] (Lord Hope), [102] (Lord Steyn), [159] (Lady Hale). See also *Axa General Insurance Ltd and Others v The Lord Advocate* [2011] UKSC 46, [2012] AC 868.

9 The UK Withdrawal from the European Union (Legal Continuity) (Scotland) Bill [2018] UKSC 64, [2019] AC 1022 [41].

10 Martin Loughlin and Stephen Tierney, ‘The Shibboleth of Sovereignty’ (2018) 81 MLR 986, 1015–16.

11 Brendan O’Leary, ‘The Character of the 1998 Agreement: Results and Prospects’ in R. Wilford (ed), *Aspects of the Belfast Agreement* (OUP 2001) 66.

12 Edmund Burke, *Reflections on the Revolution in France* (first published 1790, Dent 1910).

13 Michael Oakeshott, *Rationalism in Politics* (Methuen 1962).

14 From JAG Griffith, ‘The Political Constitution’ (1979) 42 MLR 1 to <https://judicialpowerproject.org.uk/>.

15 The House of Commons Political and Constitutional Reform Committee, *A New Magna Carta?* (HC 2014–15, 463) 24.

16 Roger Scruton, *A Political Philosophy* (Continuum 2006) vii–viii.

17 Vernon Bogdanor, *The New British Constitution* (Hart Publishing 2009) ix.

18 Home Office, *Rights Brought Home: The Human Rights Bill* (Cm 3782, 1997).

19 Roger Masterman, *The Separation of Powers in the Contemporary Constitution* (CUP 2011).

20 J Baker, ‘The Unwritten Constitution of the United Kingdom’ (2013) 15 Ecclesiastical Law Journal 4, 10.

21 Earlier proposals for a written constitution are limited to Jeremy Bentham, *Constitutional Code*, vol I (F Rosen and JH Burns ed, first published 1830, Clarendon Press 1983).

22 Lord Hailsham, ‘Elective Dictatorship’, Richard Dimbleby Lecture (BBC 1976); Lord Hailsham, *The Dilemma of Democracy: Diagnosis and Prescription* (Collins 1978). Lord Hailsham did not pursue this project as Lord Chancellor (1979–87), and by 1991 was defending the flexibility of the unwritten constitution: Ferdinand Mount, *The British Constitution Now: Recovery or Decline?* (Heinemann 1992) 3.

23 Leslie Scarman, *English Law—The New Dimension*, Hamlyn Lectures (Stevens & Sons 1974); Lord Scarman, *‘The Shape of Things to Come’: The Shape and Future Law and Constitution of the United Kingdom*, Radcliffe Lectures (University of Warwick 1989); Leslie Scarman, *Why Britain Needs a Written Constitution*, Fourth Sovereignty Lecture (Charter 88 Trust 1992).

24 Gordon Brown, *Constitutional Change and the Future of Britain*, First Sovereignty Lecture (Charter 88 Trust 1992).

25 HC Deb 10 June 2009, col 798; Labour Party Manifesto 2010, which pledged to create ‘an All Party Commission to chart a course towards a Written Constitution’ (para 9:3).

26 IPPR, *A Written Constitution for the United Kingdom* (Mansell 1991).

27 Tony Benn and Andrew Hood, *Common Sense: New Constitution for Britain* (Hutchinson 1993).

28 Vernon Bogdanor, Tarunabh Khaitan and Stefan Vogenauer, ‘Should Britain Have A Written Constitution?’ (2007) 78 Political Quarterly 499.

29 Gordon (n 5).

30 Stephen Hockman and others, ‘Towards a Codified Constitution’ (2010) 7(1) Justice.

31 Robert Blackburn, ‘Enacting a Written Constitution for the United Kingdom’ (2015) 36 Stat LR 1; Andrew. Blick, *Beyond Magna Carta: A Constitution for the United Kingdom* (Hart Publishing 2015).

32 HCPCRC (n 15).

33 <http://lsedesignunit.com/theconstitution/offline/download.pdf> accessed 31 July 2020.

34 Lady Hale, ‘The UK Constitution on the Move’, The Canadian Institute for Advanced Legal Studies’ Cambridge Lectures (7 July 2017). In his fifth 2019 Reith Lecture called ‘Shifting the Foundations’, former UK Supreme Court Justice Jonathan Sumption argued against the UK adopting a written constitution: J Sumption, *Trials of the State: Law and the Decline of Politics* (Profile Books 2019).

35 ‘Do We Need a Written Constitution?’, debate between Prof Sionaidh Douglas-Scott and Prof Nick Barber at the Constitution Unit, UCL, 28 November 2019; S Douglas-Scott and A Tomkins, ‘Does Britain Need a Proper Constitution?’ *Prospect Magazine* (2 April 2019); ‘Do We Need A Written Constitution’ (n 5).

36 BBC Radio 4, ‘Britain’s Constitutional Dilemma: Who Now Runs the Country?’ (26 September 2019); Channel 4, ‘Does Britain Need a Written Constitution after Brexit?’ (29 October 2019).

37 From ‘Britain’s Unwritten Constitution Suddenly Looks Fragile’ *New York Times* (31 August 2019) to ‘Britain Doesn’t Need a Written Constitution’ *GQ Magazine* (20 February 2020).

38 In August 2020, the Scottish Fabians, a centre-left think tank, proposed a new Act of Union, with the aim of codifying ‘a stable constitutional framework for the UK’ <http://scottish.fabians.org.uk/a-new-act-of-the-union> accessed November 2020. In February 2021, a report commissioned by the Labour Party, ‘Remaking the British State: For the Many Not the Few’ (Leader of the Opposition’s Office 2021) argued for a codified constitution as the new supreme law that would limit the law-making powers of Parliament and empower the courts to strike down unconstitutional legislation.

39 Bruce Ackerman, ‘Why Britain Needs a Written Constitution—and Can’t Wait for Parliament to Write One’ (2018) 89 Political Quarterly 584; Jeff King, ‘The Democratic Case for a Written Constitution’ [2019] CLP 1; Vernon Bogdanor, *Beyond Brexit: Towards a British Constitution* (IB Tauris 2019); B Dickson, *Writing the United Kingdom Constitution* (Manchester University Press 2019); Brian Christopher Jones, *Constitutional Idolatry and Democracy: Challenging the Infatuation with Writtenness* (Edward Elgar 2020).

40 Noted by R Hazell, ‘Do We Need a Written Constitution?’, debate at the Constitution Unit, UCL (28 November 2019).

41 Heinz Klug, ‘Constitution Making and Social Transformation’ in D Landau and H Lerner (eds), *Comparative Constitution Making* (Edward Elgar 2019) 47.

42 Giovanni Sartori, ‘Constitutionalism: A Preliminary Discussion’ (1962) 56 American Political Science Review 853, 855.

43 House of Lords Constitution Committee, *Reviewing the Constitution: Terms of Reference and Method of Working* (HL 2001–02, 11) para 20.

44 The HCPCRC Written Constitution (n 15) confirms Parliament’s exclusive powers to legislate ‘without limitation’ on all matters, including the Constitution (art 17). The Oxford student draft constitution (n 28) also preserves supremacy (at 502). The situation is unclear in the IPPR Constitution (n 26): on the one hand, ‘any law… that is inconsistent with this Constitution ceases to have effect to the extent of the inconsistency’ (art 1(4)), but on the other ,‘Parliament has exclusive powers to make laws with respect to … the Constitution’ (art 53.1). Gordon (n 5) would subject parliamentary legislation to the Constitution (art 11). Article 7.7 of the LSE’s Constitution (n 33) grants courts the power of constitutional review: ‘In the case of conflicts between the wording of this Constitution and ordinary statutes, the Constitution shall have precedence and prevail.’

45 HCPCRC (n 15) 284.

46 Jon Elster, ‘Forces and Mechanisms in the Constitution-Making Process’ (1995) 45 Duke LJ 364.

47 Klug (n 41) 48–9.

48 IPPR (n 26) 9.

49 Gordon (n 5) 6.

50 Blackburn (n 31) 5. Professor Blackburn was the lead author of the HCPCRC’s three constitutional models.

51 IPPR (n 26) 10.

52 Gordon (n 5) 7.

53 Elazar (n 6).

54 Blackburn (n 31) 4.

55 ibid 5.

56 Ipsos Mori, ‘Audit of Political Engagement 5: The 2008 Report with a Special Focus on the Constitution’ (Hansard Society 2008).

57 YouGov/Unlock Democracy Survey Results, 15–16 November 2018.

58 ComRes/Daily Express poll on 4 December 2018.

59 House of Commons Political and Constitutional Reform Committee, *A New Magna Carta?* (HC 2014–15, 599) 21.

60 Sartori (n 42) 861.

61 <www.constituteproject.org/constitution/United_Kingdom_2013.pdf>.

62 James Melton, Christine Stuart and Daniel Helen, ‘To Codify or Not to codify? Lessons from Consolidating the United Kingdom’s Constitutional Statutes’ (The Constitution Unit, UCL, March 2015) 4.

63 HCPCRC (n 15) 19.

64 HCPCRC (n 59) 17.

65 Sartori (n 42) 855.

66 ibid 854. See also The Federalist Papers No 47.

67 Stephen Holmes, *Passions and Constraint: On the Theory of Liberal Democracy* (University of Chicago Press 1995) 5–7, 161–4; John Searle, *Speech Acts: An Essay in the Philosophy of Language* (CUP 1997) 33–6.

68 Niklas Luhmann, *Law as a Social System* (KA Ziegert tr, OUP 2004) 410.

69
*The Governance of Britain* (Cm 7170, 2007) paras 204–10.

70 Commission on a Bill of Rights, ‘Do We Need a UK Bill of Rights’ (August 2011) para 26; see also the speech by the then Lord Chancellor and Secretary of State for Justice, Jack Straw, ‘Towards a Bill of Rights and Responsibilities’ (21 January 2008).

71
*The Governance of Britain* (n 69) para 210.

72 HC and HL Joint Committee on Human Rights, *A Bill of Rights for the UK?* (2007–08, HL 165–I, HC 150–I) 235.

73 ibid 52–3; see also IPPR (n 26) art 27.

74 The Universal Declaration of Human Rights, 1948.

75 PM Gordon Brown speech at Liberty (25 October 2007).

76 Jürgen Habermas, ‘Kant’s Idea of Perpetual Peace: At Two Hundred Years’ Historical Remove’ in C Cronin and P De Greiff (eds), *The Inclusion of the Other: Studies in Political Theory* (MIT Press 1998) 191.

77 HC and HL Joint Committee on Human Rights (n 72) para 34. See further TM Scanlon, ‘Rights, Goals and Fairness’ in J Waldron (ed), *Theories of Rights* (OUP 1984) 137–52.

78 Jürgen Habermas, ‘Constitutional Democracy: A Paradoxical Union of Contradictory Principles?’ (2001) 29 Political Theory 766, 779.

79 ibid 767, 779.

80 Blick (n 31) 289.

81 K Marx, ‘The Eighteenth Brumaire of Louis Bonaparte’ in D Fernbach (ed), *Surveys from Exile: Political Writings*, vol 2 (Penguin Books 1973) 146.

82 Tom Ginsburg, Daniel Rockmore and Nick Foti, ‘We the Peoples: The Global Origins of Constitutional Preambles’ (2014) 46 George Washington International Law Review 101.

83 Although the HCPCRC (n 15) para14 recommends that the constitution ‘should belong to the people of the United Kingdom’, none of the three models contain a reference to popular sovereignty. Nor does the draft constitution in Bogdanor, Khaitan and Vogenauer (n 28) 506.

84 Thomas Paine, *The Rights of Man* (first published 1791, Viking Penguin 1984) 71.

85 IPPR (n 26) Preamble; LSE Institute of Public Affairs, *The Constitution* (2015) (n 33): ‘We the people of all the nations in the United Kingdom of Great Britain and Northern Ireland being joined together in common purpose and in mutual respect, hereby affirm the permanent living principles of our democracy and the rights and responsibilities of all persons therein’. See also Gordon (n 5) 45: clause 2 provides that ‘National sovereignty shall vest in the people, who shall exercise that sovereignty through delegation of the powers as set out in this Constitution to their representatives and through the other provisions of this Constitution’.

86 I develop the outline provided in Lars Vinx, ‘The Incoherence of Strong Popular Sovereignty’ (2013) 11 ICON 101, 102–3.

87 Albert Weale, *The Will of the People: A Modern Myth* (Polity Press 2018).

88 Martin Loughlin and Neil Walker, ‘Introduction’ in Loughlin and Walker (eds), *The Paradox of Constitutionalism: Constituent Power and Constitutional Form* (OUP 2007) 2.

89 GWF Hegel, *Elements of the Philosophy of Right* (CUP 1991) § 268.

90 Prime Minister’s letter to Donald Tusk triggering Article 50, 29 March 2017 <www.gov.uk/government/publications/prime-ministers-letter-to-donald-tusk-triggering-article-50>.

91 Benedict Anderson, *Imagined Communities: Reflections on the Origin and Spread of Nationalism* (Verso 2006).

92 See generally ‘Citizenship and Belonging: What Is Britishness?’ (Commission for Racial Equality, 2005).

93 Peter Goodrich and Yifat Hachamovitch, ‘Time Out of Mind: An Introduction to the Semiotics of Common Law’ in P Fitzpatrick (ed), *Dangerous Supplements: Resistance and Renewal in Jurisprudence* (Pluto 1991) 168.

94 Jean-Jacques Rousseau, *The Social Contract* (first published 1762, Penguin 1968) Book I, ch 5.

95 ibid Book IV, ch 2.

96 ibid Book I, ch 5.

97 David Rosenfeld, ‘Rousseau’s Unanimous Contract and the Doctrine of Popular Sovereignty’ (1987) 8 History of Political Thought 83.

98 Jean-Jacques Rousseau, *Political Writings. Containing the Social Contract, Considerations on the Government of Poland, Constitutional Project for Corsica, Part I*. (University of Wisconsin Press 1986) 215; Melissa Schwartzberg, ‘Rousseau on Fundamental Law’ (2003) 51 Political Studies 387.

99 Rousseau, (n 98) 215.

100 Immanuel Kant, ‘On the Common Saying: “This May be True in Theory, but It Does Not Apply in Practice”’ in *Kant: Political Writings*, 2nd edn (first published 1793, CUP 1991) 79.

101 ibid 77, 79.

102 Immanuel Kant, *The Metaphysics of Morals* (CUP 1991) para 45.

103 ibid para 46.

104 Hans Kelsen, *Vom Wesen und Wert der Demokratie* (Mohr 1920) 14–25.

105 Vinx (n 86) 103.

106 Norberto Bobbio, *The Future of Democracy* (Polity Press 1987) 65.

107 Chris Thornhill, ‘Constitution Making and Constitutionalism in Europe’ in Landau and Lerner (n 41) 445; Chris Thornhill, ‘On Misunderstanding States: The Transnational Constitution in the National Constitution’ (2018) 16 ICON 1186, 1194.

108 Habermas, ‘Constitutional Democracy’ (n 78) 767; Jürgen Habermas, *Between Facts and Norms: Contributions to a Discourse Theory of Law and Democracy* (MIT Press 1996) 121–2.

109 Jürgen Habermas, ‘Remark’s on Dieter Grimm’s “Does Europe Need a Constitution?”’ in P Gowan and P Anderson (eds), *The Question of Europe* (Verso 1997) 262.

110 Michael Zander, *A Bill of Rights?* (Sweet & Maxwell 1975); RM Dworkin, *A Bill of Rights for Britain: Why British Liberty Needs Protection* (Chatto & Windus 1990).

111 Neil Walker, ‘Our Constitutional Unsettlement’ [2014] PL 529.

112 George Orwell, ‘England, Your England’ *Selected Essays* (Penguin Books 1957) 72: ‘we call our islands by no less than six different names, England, Britain, Great Britain, the British Isles, the United Kingdom and, in very exalted moments, Albion’.

113 Under this provision, Northern Ireland remains part of the UK unless and until ‘a majority of the people of Northern Ireland voting in a poll’ express a wish to form part of a united Ireland. See also Belfast Agreement, 10 April 1998, art 1(ii); HM Government, *New Withdrawal Agreement and Political Declaration* (19 October 2019); Ireland/Northern Ireland Protocol, art 1(1). In January 2021, a poll by LucidTalk for the *Sunday Times* found that a majority (51%) of those surveyed in Northern Ireland would support a referendum in the next five years. The same poll found that 47% were in favour of remaining part of the UK, with 42% in favour of a united Ireland, which falls short of the criterion in s 1 of the Northern Ireland Act 1998.

114 The Scottish National Party’s 2019 General Election manifesto stated the party’s intention to hold a second independence referendum. Having won a majority of Scotland’s seats, First Minister Nicola Sturgeon claimed to have a ‘cast-iron mandate’. To that end, she sent an official request to the Prime Minister in December 2019 which was rejected in January 2020.

115 Welsh Government, *Reforming Our Union: Shared Governance in the UK* (2019) Proposition 1.

116 ‘Welsh Independence Referendum “Before 2030” Plaid Leader Says’ (*BBC Online*, 4 October 2019). According to the Savanta ComRes/ITV Welsh Barometer Poll in March 2021, support for Welsh independence has gradually increased to 39% of those surveyed—the highest level of support for Welsh independence ever recorded. A clear majority (55%) supports further devolution of powers to the Welsh Parliament: ICM Unlimited/BBC Wales—St David’s Day Poll 2020. See also the non-party campaign for independence <www.yes.cymru>, whose membership jumped from 2000 in early 2020 to over 17,000 in early 2021: BBC News, ‘Yes Cymru: Independence “Not Possible” Without Political Change’ (17 January 2021).

117 AV Dicey, *Introduction to the Study of the Law of the Constitution* (10th edn, Macmillan 1959) 37. See also the critique in A de Tocqueville, *Democracy in America* (Sever & Francis 1863) 126: ‘In England, the constitution may change continually; or rather, it does not in reality exist; the Parliament is at once a legislative and a constituent assembly.’

118 Jon Elster, ‘Legislatures as Constituent Assemblies’ in RW Bauman and T Kahana (eds), *The Least Examined Branch: The Role of Legislatures in the Constitutional State* (CUP 2006).

119 HCPCRC (n 15).

120 House of Commons Political and Constitutional Reform Committee, *Do We Need a Constitutional Convention for the UK?* (HC 2012–13, 371).

121 <https://services.parliament.uk/bills/2015-16/constitutionalconventionno2.html> accessed in August 2020.

122 Ackerman (n 39) 588.

123 ibid 588.

124 King (n 39) 5.

125 ibid 27.

126 ibid 8.

127 ibid 5.

128 Ackerman (n 39) 584, 588–9.

129 See s 28(7) of the Scotland Act, s 107(5) of the Government of Wales Act 2006 and s 5(6) of the Northern Ireland Act 1998.

130 Article 1 of the Belfast/Good Friday Agreement 1998 states that the right of self-determination for ‘the people of the island of Ireland alone’ is to be exercised ‘on the basis of consent’. The Northern Ireland Act 1998, s 1 states that Northern Ireland ‘shall not cease’ in its entirety to be part of the UK ‘without the consent of a majority of the people of Northern Ireland voting in a poll held for the purposes’.

131 Scotland Act 2016, s 1; Wales Act 2017, s 1.

132 Vlad Constantinesco, ‘L’Union européene: par le droi ou vers le politique? (ad augusta per augusta?)’ in G Duprat (ed), *L’Union européene, droit, politique, démocratie* (Presses universitaires de France 1996) 186; Jürgen Habermas, *The Crisis of the European Union: A Response* (Polity Press 2012) 28–37; Markus Patberg, ‘Introduction: The EU’s Pouvoir Constituant Mixte—Exploring the Systematic Potential of an Innovative Category’ (2017) 55 JCMS 165, and articles by Habermas, Peter Niesen, Jelena von Achenbach, Markus Patberg and Klaus Günther in the same issue.

133 Habermas, *The Crisis of the European Union* (n 132) 37.

134 L Moreno, ‘Scotland, Catalonia, Europeanization and the “Moreno Question’” (2006) 54 Scottish Affairs 1.

135 YouGov/BBC English Identity Survey Results, March 2018.

136 YouGov/BBC Attitudes to Scottishness Survey Results, April 2018.

137 ICM Unlimited/BBC Wales—St David’s Day Poll 2019.

138 Agreement between the Government of the United Kingdom of Great Britain and Northern Ireland and the Government of Ireland, 10 April 1998, art 1(iv).

139 45% in the Northern Ireland Life and Times Survey, Political Attitudes, 2019; 47% in the LucidTalk/YouGov/BBC, Northern Ireland Tracker Poll, May 2018.

140 See further Kevin McNicholl, Clifford Stevenson and John Garry, ‘How the “Northern Irish” National Identity Is Understood and Used by Young People and Politicians’ (2019) 40 Political Psychology 487.

141 Günter Frankenberg, ‘Constitutions as Commodities: Notes on a Theory of Transfer’ (2013) 4 Comparative Law Review 1, 2.

142 Neil MacCormick, *Questioning Sovereignty* (OUP 1999) 104.

143 Hanna Lerner and Asli Ü Bali, ‘Constitutional Design without Constitutional Moments: Lessons from Religiously Divided Societies’ (2016) 49 Cornell Int’l LJ 227, 306.

